# Allometry and Ecology of the Bilaterian Gut Microbiome

**DOI:** 10.1128/mBio.00319-18

**Published:** 2018-03-27

**Authors:** Scott Sherrill-Mix, Kevin McCormick, Abigail Lauder, Aubrey Bailey, Laurie Zimmerman, Yingying Li, Jean-Bosco N. Django, Paco Bertolani, Christelle Colin, John A. Hart, Terese B. Hart, Alexander V. Georgiev, Crickette M. Sanz, David B. Morgan, Rebeca Atencia, Debby Cox, Martin N. Muller, Volker Sommer, Alexander K. Piel, Fiona A. Stewart, Sheri Speede, Joe Roman, Gary Wu, Josh Taylor, Rudolf Bohm, Heather M. Rose, John Carlson, Deus Mjungu, Paul Schmidt, Celeste Gaughan, Joyslin I. Bushman, Ella Schmidt, Kyle Bittinger, Ronald G. Collman, Beatrice H. Hahn, Frederic D. Bushman

**Affiliations:** a Department of Microbiology, University of Pennsylvania, Philadelphia, Pennsylvania, USA; b Department of Medicine, University of Pennsylvania, Philadelphia, Pennsylvania, USA; c Department of Ecology and Management of Plant and Animal Resources, Faculty of Sciences, University of Kisangani, Kisangani, Democratic Republic of the Congo; d Leverhulme Centre for Human Evolutionary Studies, University of Cambridge, United Kingdom; e Projet Primates France, Centre de Conservation pour Chimpanzés, Faranah, Republic of Guinea; f Lukuru Wildlife Research Foundation, Tshuapa-Lomami-Lualaba Project, Kinshasa, Democratic Republic of the Congo; g Department of Human Evolutionary Biology, Harvard University, Cambridge, Massachusetts, USA; h School of Biological Sciences, Bangor University, Bangor, United Kingdom; i Department of Anthropology, Washington University in St. Louis, St. Louis, Missouri, USA; j Lester E. Fisher Center for the Study and Conservation of Apes, Lincoln Park Zoo, Chicago, Illinois, USA; k Tchimpounga Chimpanzee Rehabilitation Center, Jane Goodall Institute, Pointe Noire, Republic of Congo; l Department of Anthropology, University of New Mexico, Albuquerque, New Mexico, USA; m Department of Anthropology, University College London, London, United Kingdom; n Department of Natural Sciences, Liverpool John Moores University, Liverpool, United Kingdom; o Sanaga-Yong Chimpanzee Rescue Center, IDA-Africa, Portland, Oregon, USA; p Gund Institute for Environment, Rubenstein School for Environment and Natural Resources, University of Vermont, Burlington, Vermont, USA; q Division of Gastroenterology, Perelman School of Medicine, University of Pennsylvania, Philadelphia, Pennsylvania, USA; r Tulane National Primate Research Center, Tulane University Health Science Center, Covington, Louisiana, USA; s Thomas Jefferson University, Philadelphia, Pennsylvania, USA; t Southeast Fisheries Science Center, National Oceanic and Atmospheric Administration Fisheries Service, Panama City, Florida, USA; u Gombe Stream Research Centre, The Jane Goodall Institute, Kigoma, Tanzania; v Department of Biology, University of Pennsylvania, Philadelphia, Pennsylvania, USA; w Division of Gastroenterology, Hepatology, and Nutrition, The Children’s Hospital of Philadelphia, Philadelphia, Pennsylvania, USA; New York University School of Medicine

**Keywords:** bacteria, bilateria, microbiome, microbiota, neutral model, species-area

## Abstract

Classical ecology provides principles for construction and function of biological communities, but to what extent these apply to the animal-associated microbiota is just beginning to be assessed. Here, we investigated the influence of several well-known ecological principles on animal-associated microbiota by characterizing gut microbial specimens from bilaterally symmetrical animals (*Bilateria*) ranging from flies to whales. A rigorously vetted sample set containing 265 specimens from 64 species was assembled. Bacterial lineages were characterized by 16S rRNA gene sequencing. Previously published samples were also compared, allowing analysis of over 1,098 samples in total. A restricted number of bacterial phyla was found to account for the great majority of gut colonists. Gut microbial composition was associated with host phylogeny and diet. We identified numerous gut bacterial 16S rRNA gene sequences that diverged deeply from previously studied taxa, identifying opportunities to discover new bacterial types. The number of bacterial lineages per gut sample was positively associated with animal mass, paralleling known species-area relationships from island biogeography and implicating body size as a determinant of community stability and niche complexity. Samples from larger animals harbored greater numbers of anaerobic communities, specifying a mechanism for generating more-complex microbial environments. Predictions for species/abundance relationships from models of neutral colonization did not match the data set, pointing to alternative mechanisms such as selection of specific colonists by environmental niche. Taken together, the data suggest that niche complexity increases with gut size and that niche selection forces dominate gut community construction.

## IMPORTANCE

The intestinal microbiome of animals is essential for health, contributing to digestion of foods, proper immune development, inhibition of pathogen colonization, and catabolism of xenobiotic compounds. How these communities assemble and persist is just beginning to be investigated. Here we interrogated a set of gut samples from a wide range of animals to investigate the roles of selection and random processes in microbial community construction. We show that the numbers of bacterial species increased with the weight of host organisms, paralleling findings from studies of island biogeography. Communities in larger organisms tended to be more anaerobic, suggesting one mechanism for niche diversification. Nonselective processes enable specific predictions for community structure, but our samples did not match the predictions of the neutral model. Thus, these findings highlight the importance of niche selection in community construction and suggest mechanisms of niche diversification.

## INTRODUCTION

Classical ecology provides extensive theories for investigating community dynamics that are beginning to be applied to sequence-based studies of microbes. For example, species-area relationships (1) have been called “one of community ecology’s few genuine laws” (2, 3). According to this idea, for islands located at the same distance from a mainland source, the larger islands accumulate the greater number of species, because (i) the larger population sizes that are possible only on larger islands reduce the chances of stochastic fluctuations leading to extinction and (ii) larger islands have a greater number of niches, supporting more different types of residents. Species-area relationships have been investigated previously for the gut microbiota but with inconsistent results. One study using published values from 16S rRNA gene sequencing studies of rRNA gene segments led to the conclusion that the mammalian gut microbiota did not show greater richness in intestines of larger organisms (4). Another study, which used a DNA fingerprinting method to characterize bacterial gut communities, concluded that richness indeed increased for animals of larger sizes (5).

Another ecological framework, Hubbell’s neutral model, makes specific predictions for neutral colonization processes that can then be compared to data to interrogate assembly mechanisms. Hubbell’s neutral theory models construction of a population via migration from a source population using information on birth and death rates, migration rates, and abundances while assuming all organisms are equally fit colonists (6). Neutral theory provides quantitative predictions for the relative abundances of community members, which can then be compared to empirical data to assess whether neutral theory provides a compelling explanation of observations (7). In the absence of a compelling fit, other processes, typically involving niche selection, can be hypothesized to be more likely. In a recent study, human microbiome specimens were tested and usually found to diverge from the predictions of neutral theory (8), but this has not been investigated for a larger number of animals.

We thus sought to investigate microbial community structure as a window into assembly processes and so studied a wide set of members of the *Bilateria*—the bilaterally symmetrical clade of animals that includes protostomes and deuterostomes. We focused on intestinal bacterial communities to access a consistent sample type and to interrogate an organ system relevant to human and veterinary medicine. In all, we sampled gut contents from 10 classes of animals, representing 64 species. We purified DNA from feces or dissected gut tissue from species that ranged in size from bedbug (0.009 g) to right whale (72,000,000 g), spanning almost 10^9^ g in body size. In this analysis, we assumed that overall organismic size is a viable surrogate for gut size, though it is clearly an approximation (9). To analyze bacterial communities, we amplified and sequenced 16S rRNA gene segments, allowing quantification of the richness and diversity of bacterial communities. Samples were rigorously vetted to control for artifacts due to low biomass in starting samples (10, 11). We also compared data from several published studies (12–19), yielding 1,098 total samples.

We found that gut microbial community structure was associated with host phylogeny. Species-area analysis showed a positive correlation between organismic size and gut microbial species richness. Further analysis showed that community structure did not fit predictions of neutral assembly models, with the total data implying a prominent role for niche selection in assembly of gut microbial communities.

## RESULTS

### Experimental strategy.

To compare gut microbial populations among bilaterians of different sizes, we collected 444 specimens of feces or dissected intestines (see Table S1 in the supplemental material). Because some samples contained host tissue, we used 16S rRNA gene primers for amplification and sequencing, so that our sequencing effort queried bacterial colonists and not sequences from the host. Amplification was carried out with primers targeting the V1V2 region of the 16S rRNA gene, which has been used previously for phylogenetic comparisons of gut microbiota (17, 20).

10.1128/mBio.00319-18.7 Table S1  Samples studied and associated metadata. Download Table S1, XLSX file, 0.1 MB. Copyright © 2018 Sherrill-Mix et al. 2018 Sherrill-Mix et al. This content is distributed under the terms of the Creative Commons Attribution 4.0 International license.

Low amounts of starting template are problematic because reagent contamination can become a significant proportion of the total (10, 11). We thus carried out 16S quantitative PCR (qPCR) analysis of all samples studied and analyzed only samples where at least 1,000 16S rRNA gene copies could be introduced into PCRs for library preparation. Samples with fewer than 1,000 reads after quality filtering were also excluded. All DNA isolation and sequencing steps were carried out side by side with negative controls, allowing us to query contamination from reagents, dust, and other sources (see Fig. S1 in the supplemental material). Samples with extensive representation of lineages found in negative controls typically contained low starting copy numbers of bacterial DNA and were among those excluded by our qPCR criteria. This yielded 265 samples from 10 classes, representing 64 species of *Bilateria* (Table S1) (Fig. S1A).

10.1128/mBio.00319-18.1 FIG S1  (A) Taxonomic breakdown of bilaterian host samples reported in this study. (B) Summary of the average numbers of reads per sample for various sample types. Four samples (3 animal and 1 negative control) with 0 reads are not shown. Naming of control samples is as follows: dissection, washes of instruments used prior to dissection; extraction, mock purification of DNA-free water; negControl, extractions with no added sample; posControl, synthetic DNAs used as methodological positive controls as described in reference 11. (C) Summary of the number of reads assigned to the most abundant phyla identified in the bilaterian gut samples. (D) Sampling sites for ape fecal samples. Field sites are shown in relation to the ranges of the four subspecies of the common chimpanzee (inset: *Pan troglodytes verus*, black; *Pan troglodytes ellioti*, purple; *Pan troglodytes troglodytes*, magenta; *Pan troglodytes schweinfurthii*, blue) and western lowland (*Gorilla gorilla gorilla*, red stripe) and eastern lowland (*Gorilla beringei graueri*, cyan stripe) gorilla, as well as the bonobo (*Pan paniscus*, orange), in sub-Saharan Africa. Field sites are labeled by a two-letter code, with circles, squares, and hexagons identifying locations where fecal samples were collected from chimpanzee species, gorilla species, and both groups of species, respectively. Triangles denote ape rescue centers, and ovals indicate bonobo field sites. CC, center for chimp conservation (sanctuary); GA, Gashaka; MT, Minta; DG, Diang; SY, Sanaga-Yong (sanctuary); BQ, Belgique; GT, Goalougo triangle; UB, Ubangi; LG, Lingunda; KR, Kokolopori; LK, Lui-Kotal; IK, Ikela; TL2, Tshuapa-Lomami-Lualaba; BI, Babingi; PA, Parisi; OP, Opienge; KE, Kasese; TC, Tchimpounga (sanctuary); GM, Gombe national park; LU, Lubutu; UG, Ugalla. The Chimfunshi (sanctuary) site (CF) in Zambia is not indicated. Download FIG S1, PDF file, 1 MB. Copyright © 2018 Sherrill-Mix et al. 2018 Sherrill-Mix et al. This content is distributed under the terms of the Creative Commons Attribution 4.0 International license.

**FIG 1:**
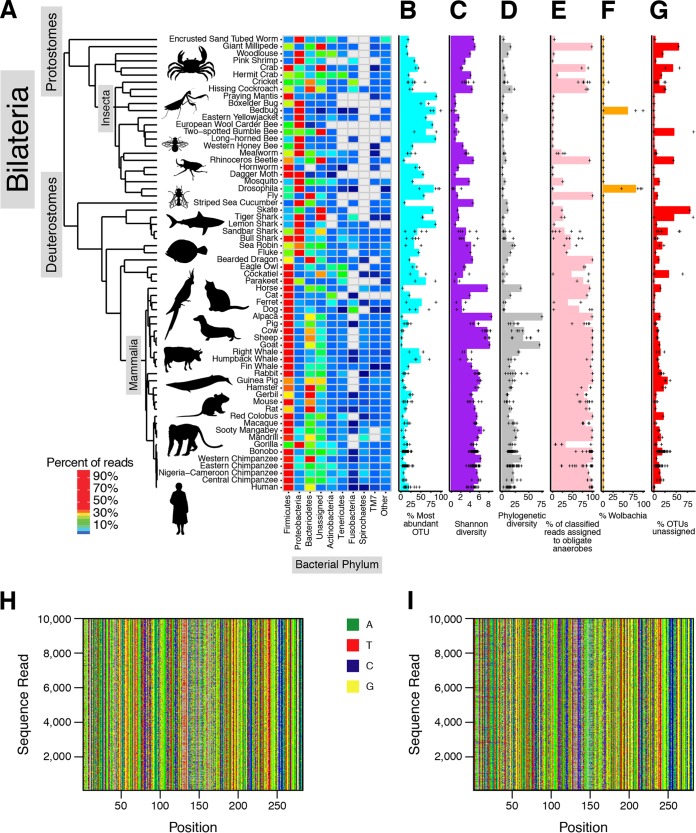
Gut microbiota of the *Bilateria*. The figure summarizes several forms of analysis of the 16S rRNA gene tag sequence data. Host species are indicated by their common names—formal genus and species designations are in Table S1. (A) The proportional abundance of the most abundant bacterial phyla averaged by species. (B) Bar graphs summarizing the percentages of reads assigned to the proportionally most abundant OTU in each species. The values were averaged for each species (bar), and values for individual samples are indicated by plus (+) symbols (the graph format repeats in panels C to G). (C) Bar graph summarizing Shannon diversity for the microbial communities of the various species. (D) Bar graph summarizing phylogenetic diversity for the microbial communities of the various species. (E) Bar graph summarizing the proportion of annotated obligate anaerobic bacteria for the various species. (F) Bar graph summarizing the percentages of reads annotated as *Wolbachia* for the various species. (G) Bar graphs summarizing the percentages of OTUs that were unable to be assigned to references in the Greengenes database. (H and I) Alignments of a 10,000-read random subset of assigned (H) and unassigned (I) sequence reads showing positions with greater than 10% non-gap sequences, emphasizing that the unassigned reads resemble the assigned reads in sequence.

We expected that bacteria in little-studied bilaterian species would be disproportionately difficult to assign to genera and species, so bacterial lineages were characterized as operational taxonomic units (OTUs) based on sequence and not as phylogenetic assignments based on database references. Sequence reads were condensed into clusters of 97% identity using UCLUST (Table S2). Phylogenetic placement was assessed where possible by comparison to the Greengenes database (Fig. 1A). On average, each specimen was characterized by 104,998 sequence reads (Fig. S1B and C), yielding on average 3,551 OTUs. Numbers ranged from 21 OTUs in a sample (boxelder bug) to 26,991 OTUs (Jersey cow).

10.1128/mBio.00319-18.8 Table S2  OTUs in bilaterian samples and negative controls. Download Table S2, XLSX file, 8.4 MB. Copyright © 2018 Sherrill-Mix et al. 2018 Sherrill-Mix et al. This content is distributed under the terms of the Creative Commons Attribution 4.0 International license.

We also collected additional data from previously published studies on the microbiome of insects (12), birds (13), primates (14), myrmecophagous mammals (15), fish (16), and other mammals (17–19). Combining newly generated data with published data yielded 1,098 specimens for analysis.

### Bacterial communities in the *Bilateria.*

Figure 1 summarizes the samples newly studied here and their bacterial communities (similar summaries for the published data sets are presented in Fig. S2A to J). Overall, 64 species were queried, which included broad sampling of both the protostomes and deuterostomes. The predominant bacterial phyla are summarized in the heat maps in Fig. 1A, organized by the host phylogeny (a detailed summary of sample phylogeny is presented in Fig. S1A). Among the vertebrates, *Mammalia* were dominated by *Firmicutes* and *Bacteroides*, as has been reported in many studies. *Proteobacteria* and *Actinobacteria* were present to lesser extents. Birds showed high levels of colonization with *Firmicutes* and *Tenericutes*. Fish and sharks, in contrast, consistently showed high levels of colonization with levels of *Proteobacteria*. Among invertebrates, colonization by *Proteobacteria* typically predominated.

10.1128/mBio.00319-18.2 FIG S2  (A) Summary of the abundances and diversity of a gut microbiome data set from myrmecophagous mammals (15). (B) Summary of the abundances and diversity of a gut microbiome data set from birds (13). (C) Summary of the abundances and diversity of a gut microbiome data set from insects (12). The panel shows the forward direction sequence reads. (D) Summary of the abundances and diversity of a gut microbiome data set from insects (12). The panel shows the reverse-direction sequence reads. (E) Summary of the abundances and diversity of a gut microbiome data set from fish (16). (F) Summary of the abundances and diversity of a gut microbiome data set from mammals (17). (G) Summary of the abundances and diversity of a gut microbiome data set from mammals (19). (H) Summary of the abundances and diversity of a gut microbiome data set from primates (14). (I) Summary of the abundances and diversity of a gut microbiome data set from cetaceans and other mammals (18). The panel shows 454-sequenced samples. (J) Summary of the abundances and diversity of a second gut microbiome data of cetaceans and other mammals (18). The panel shows Illumina-sequenced samples. Download FIG S2, PDF file, 2.8 MB. Copyright © 2018 Sherrill-Mix et al. 2018 Sherrill-Mix et al. This content is distributed under the terms of the Creative Commons Attribution 4.0 International license.

In our data, nine phyla accounted for 87.7% of the data—in order of abundance, these were *Firmicutes*, *Bacteroidetes*, *Proteobacteria*, *Actinobacteria*, *Tenericutes*, *Spirochaetes*, *Fusobacteria*, *Cyanobacteria*, and TM7 (Fig. S1C). Twelve percent of sequences remained unassigned by comparison to the Greengenes database, emphasizing the frequency of unstudied microbial lineages in the bilaterian microbiome. These reads were most commonly found in little-studied host species, such as crab (an average of 42% of reads were not assigned), giant millipede (55%), and skate (80%). In contrast, for the well-studied case of the human microbiome (21, 22), an average of only 0.7% of reads were not classified.

### Shared and unique features of bilaterian gut communities.

We began to assess community structure by asking what proportion of the full sample was comprised of the most predominant OTU (Fig. 1B). As is discussed below, assessing the rank/abundance structure of communities provides potential insight into the process by which they were generated. A broad range of values were seen, with highly diverse communities in primates and ungulates having maximum OTU abundance ranging from only 1.6% (goat) to 18.1% (human), while many sharks and rays (3 of 5 species) and insects (10 of 17 species) showed 50% or more of the communities comprised of the most predominant OTU. The Shannon diversity index showed reciprocal behavior, as expected (Fig. 1C). Samples were further compared using phylogenetic diversity (Fig. 1D), which measures biodiversity as branch length on a phylogenetic tree. Here a strong separation was evident between protostomes, which showed low phylogenetic diversity, and deuterostomes, for which phylogenetic diversity was substantially higher (Wilcoxon rank sum *P* < 10^−6^). Related patterns could be seen in the published data sets analyzed in Fig. S2A to J, with deuterostomes typically showing higher levels of phylogenetic diversity.

Given these survey data, we could also compare levels of oxygen utilization by gut bacterial communities. Literature data were used to annotate the oxygen utilization and sensitivity of each bacterial species; values were first pooled for bacteria in a community and then for individuals within a host species, to generate an overall value for each host species (Fig. 1E; see also Fig. S3A and B). A trend was observed in which the mammals tended to have a larger fraction of obligate anaerobes in their guts whereas the smaller insects typically showed a smaller fraction of anaerobes. We return to this point below.

10.1128/mBio.00319-18.3 FIG S3  (A) Proportions of inferred oxygen utilization averaged across samples from gut bacterial communities for each species. (B) Correlation between the ratio of the reads assigned and the ratio of aerobes to obligate anaerobes and host organismic weight. Points indicate the average for each species and are colored by host phylogenetic class; the line indicates a linear regression of the ratio of aerobes to obligate anaerobes on log host weight, and shading indicates the 95% confidence interval for the regression. Download FIG S3, PDF file, 0.3 MB. Copyright © 2018 Sherrill-Mix et al. 2018 Sherrill-Mix et al. This content is distributed under the terms of the Creative Commons Attribution 4.0 International license.

Cells of many invertebrates are known to be potentially colonized by *Wolbachia*, a Gram-negative *Alphaproteobacteria* species of the *Rickettsiaceae* family. A previous sequence survey identified *Wolbachia* in multiple insect species (12) (Fig. S2C). Our sample set offered the opportunity to assess colonization broadly among the *Bilateria* (Fig. 1F). We found colonization in *Drosophila*, as has been well described, and also colonization in bed bugs and crickets. We did not detect *Wolbachia* outside the class *Insecta*.

Of particular interest is the issue of how frequently unstudied lineages were encountered. For example, recent studies of lineages from mouths of dolphins revealed unusual deep-branching lineages in host-associated communities (23). To begin to probe this issue, we asked what proportion of the lineages detected could not be assigned even to the phylum level (Fig. 1G). We identified specific host organisms that had notably high fractions of unassigned bacterial lineages (as judged by comparison to the Greengenes database), including tiger shark, skate, giant millipede, two-spotted bumble bee, and rhinoceros beetle. Analysis of these sequences indicated that despite their divergence, they were recognizable as 16S rRNA gene sequences (Fig. 1H and I). Further analysis using BLAST allowed some of these sequences to be tentatively assigned, revealing, for example, possible members of the uncommon phyla GN02 and SR1 and candidate phylum SBR1093 (Fig. S4). Analysis of published data sets showed that unassigned lineages were enriched in invertebrates and fish as well and that more fully characterized bacterial lineages were more common in mammals (Fig. S2A to J). These findings provide promising starting points for future studies targeting discovery of novel gut bacteria.

10.1128/mBio.00319-18.4 FIG S4  Abundance of some deep-branching lineages. A heat map representing proportions (species average) for divergent OTUs is shown. Divergent OTUs from the inferred phylogenetic tree were selected by finding any OTU whose ancestry contained a single branch length of at least 10% nucleotide difference. This resulted in a set of 669 OTUs (of 521,510). Read counts for 254 divergent OTUs were aggregated into 85 taxonomic assignments from Greengenes. OTUs with taxonomic assignments are labeled with their assigned phylum (indicated by "p_") concatenated to their most specific phylogenetic assignment ("c_" indicates class; "o_" indicates order; "f_" indicates family; "g_" indicates genus). The remaining 415 divergent OTUs had no taxonomic assignment in the Greengenes database and are labeled “Unassigned” (concatenated to an arbitrary identifier). Taxonomic assignments were ranked by number of distinct host species in which they were detected, and the top 24 are shown as columns in the heat map, with aggregate counts of all the remaining divergent OTUs indicated in the far right column. Download FIG S4, PDF file, 0.3 MB. Copyright © 2018 Sherrill-Mix et al. 2018 Sherrill-Mix et al. This content is distributed under the terms of the Creative Commons Attribution 4.0 International license.

### Community structure, phylogeny, and lifestyle.

**FIG 2:**
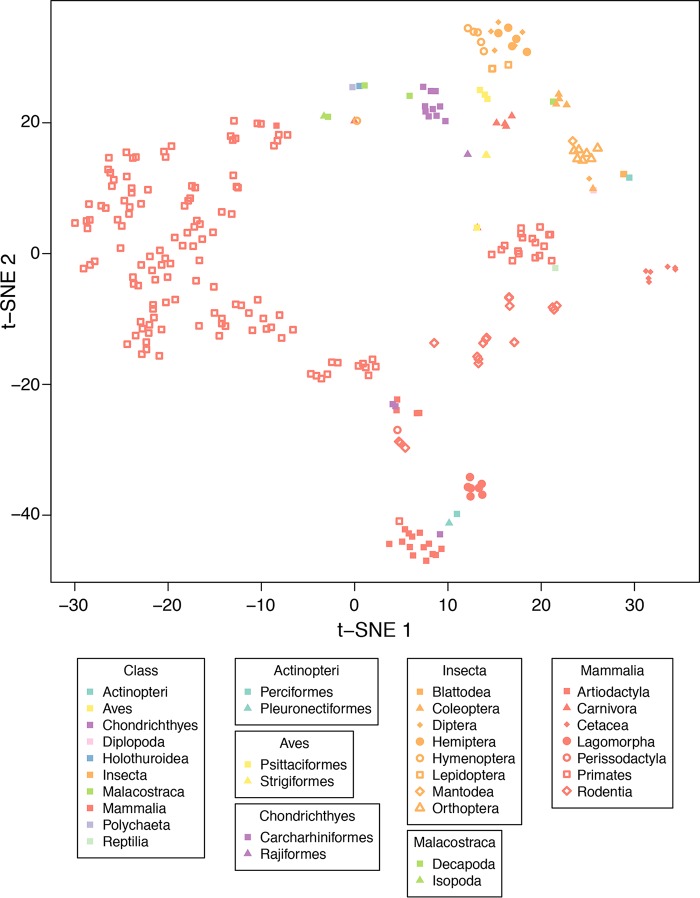
t-SNE plot of UniFrac distances. A two-dimensional representation of unweighted UniFrac distances was generated using t-distributed stochastic neighbor embedding (t-SNE). Samples are colored to indicate phylogenetic class, and orders are further broken out by point shape. Weighted and unweighted UniFrac t-SNE plots of species centroids (including species names) are shown in Fig. S5.

We next compared bilaterian gut community structures on a phylogenetic scaffold using UniFrac. In this method, two samples are compared by arranging their OTU sequences on a common tree and quantifying how much of the branch length is unique to each sample. Figure 2 summarizes data for unweighted UniFrac, which generates distances based on presence/absence information. All pairwise distances between samples were calculated, and the resulting matrix was reduced to a two-dimensional summary using t-distributed stochastic neighbor embedding (t-SNE). Sample clustering associated with host phylogeny was apparent. Such clustering was also seen when species centroids were used rather than individual samples (Fig. S5A to D). Mammals formed a broad cluster, with notable subclusters for specific clades, e.g., *Cetacea*, *Artiodactyla*, *Langomorpha*, and *Rodentia*. Primates, extensively represented in this data set, formed a broad cluster, with specific subclusters classified by species. Members of *Insecta* formed a particularly tight group, with subclusters for *Orthoptera*, *Diptera*, *Hymenoptera*, and *Coleoptera*.

10.1128/mBio.00319-18.5 FIG S5  (A) t-SNE plot of unweighted UniFrac species centroids. A two-dimensional representation of unweighted UniFrac distances between species centroids was generated using t-distributed stochastic neighbor embedding (t-SNE). Colors indicate host phylogenetic class as described for Fig. 2. (B) t-SNE plot of weighted UniFrac species centroids. A two-dimensional representation of weighted UniFrac distances between species centroids was generated using t-SNE. Colors indicate host phylogenetic class as described for Fig. 2. Download FIG S5, PDF file, 0.2 MB. Copyright © 2018 Sherrill-Mix et al. 2018 Sherrill-Mix et al. This content is distributed under the terms of the Creative Commons Attribution 4.0 International license.

We used several approaches to measure this clustering with host taxonomy. Permutational multivariate analysis of variance (PERMANOVA) testing showed that gut microbial communities clustered with host taxonomy for all host taxonomic levels queried (PERMANOVA *P* < 10^−6^ for phylum, class, order, family, genus, and species). In another analytic approach, evolutionary times of divergence among all host species were obtained from TimeTree (24), and the resulting host evolutionary distance matrix was compared to the unweighted UniFrac matrix using the Mantel test. The level of overlay of the two matrices was found to be significantly higher than would be expected by chance (Mantel *P* < 10^−6^), indicating a strong association.

Samples also partitioned by diet, although diet was commonly correlated with host phylogeny and difficult to disentangle in our data. Samples clustered by diet type (carnivore, omnivore, or herbivore) at a high level of significance (PERMANOVA *P* < 10^−6^). Even after accounting for phylum identity, the clustering was still highly significant (PERMANOVA *P* < 10^−6^). Samples from *Chondrichthyes*, carnivorous sharks and rays, clustered (PERMANOVA *P* < 10^−6^), as did samples from *Carnivora* (PERMANOVA *P* = 0.0001; *P* = 0.02 for clustering within mammals). Among the previously published whale samples (18), those from the filter-feeding whales were all dominated by *Firmicutes* (Fig. 1; see also Fig. S2I and J) and clustered seperately (PERMANOVA *P* = 0.0002) from the carnivorous toothed whales which were dominated by other phyla (Fig. S2I and J). Thus, our data are consistent with a role for diet in gut microbiota diversification, as has been suggested previously for humans (22, 25) and, broadly, for mammals (17, 26), but diet was convoluted with taxonomy in our data. Host organism weight was also significantly associated with community structure, even after accounting for host order (PERMANOVA *P* < 10^−6^).

### Species-area relationships in the bilaterian microbiome.

We next investigated gut microbial colonization from the perspective of species-area analysis. In the ecological literature, a strong pattern has been observed whereby larger islands are more species rich (assuming that each of the islands compared is the same distance from a continental source of migrants) (1–3). Here we carried out a species-area analysis of over 1,098 gut microbiome samples, including our rigorously vetted sample series. Organism weight was used to approximate gut size, allowing us to compare organisms ranging from bedbug (0.009 g) to right whale (72,000,000 g).

**FIG 3:**
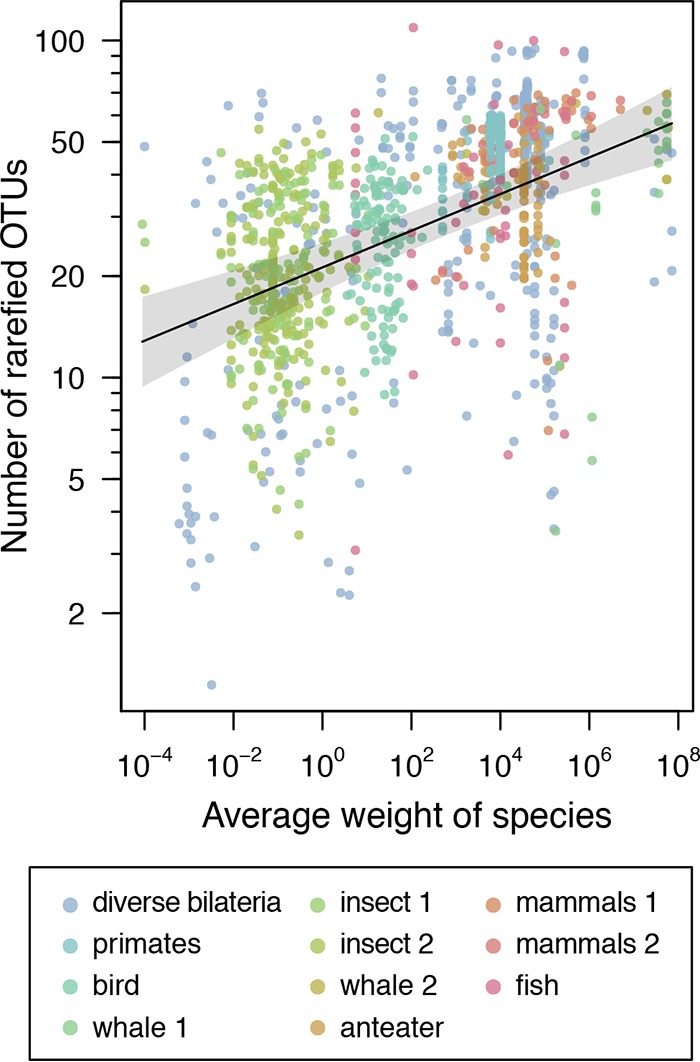
Species-area analysis. The relationship between the weight of the host organism and the number of gut bacterial OTUs found in fecal samples was estimated using a Bayesian regression model. All samples, both those newly determined here and those from previously published data sets, are included. The gray-shaded region shows the 95% credible interval for the slope. The OTU counts were normalized across sample sets as described in Materials and Methods. Origins of sample sets are as follows: diverse *Bilateria*, this work; primates, reference 14; birds, reference 13; whale 1 and 2, reference 18; insects 1 and 2, reference 12; anteaters, reference 15; fish, reference 16; mammals 1, reference 17; mammals 2, reference 19. Samples were rarefied to 100 reads each.

Modeling log bacterial richness (expected number of rarefied OTUs) as a linear function of log weight yielded a significant positive slope of 0.054 (95% credible interval of the posterior mean, 0.036 to 0.073), indicating greater richness in the larger guts (Fig. 3). This supports models in which (i) larger guts contain more niches and/or (ii) the larger microbial population sizes possible in larger guts oppose extinction due to stochastic fluctuations in numbers.

Assessment of oxygen utilization by bacterial communities showed a correlation with host body weight. Each bacterial lineage was analyzed where possible for its status as an anaerobe or aerobe, and the results were pooled for the bacterial lineages within a host individual and then for host individuals within a species (Fig. S3A). The ratio of aerobes to obligate anaerobes was significantly correlated with host weight (Fig. S3B; Pearson correlation = −0.73, *P* < 10^−6^), suggesting that the larger guts may be more anaerobic in the luminal space and that this may result in new niche creation.

### Community structure and models for community assembly.

Ecological theory describing colonization of islands in the absence of deterministic factors—the neutral model—predicts the shape of rank abundance curves, allowing our data to be interrogated to assess agreement with this prediction. According to this model, colonization is a function of immigration of individuals from a source population, together with their birth and death rates, and all potential colonists are equal. Lack of agreement between predicted and observed rank-abundance curves allows rejection of the neutral model, pointing instead to alternative theories such as selection of specific colonists by environmental niches.

**FIG 4:**
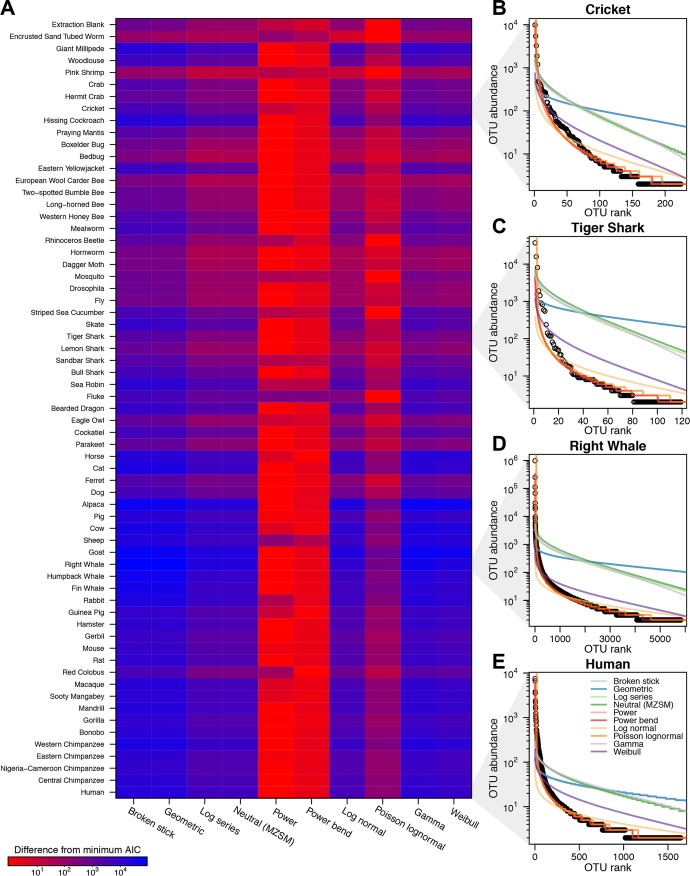
The bilaterian gut microbiota does not fit predictions of neutral assembly models. (A) The abundance of OTUs from each sample was assessed for their fits to community models (columns) using Akaike information content (AIC) and the AIC averaged within each species (rows). Within each row, the best-fitting models (i.e., those with the lowest AIC levels) appear red, with the color code showing the difference for each model from the minimum AIC. (B to E) Empirical rank-abundance curves and comparisons to model best fits are shown for single samples from human (B), right whale (C), tiger shark (D), and cricket (E).

Figure 4 shows an assessment of fits to 10 model types, including the neutral model. Comparisons based on Akaike information content showed that the neutral model fits poorly relative to other simple models of species abundance (Fig. 4A). An elaboration of the neutral model where microbiota are acquired vertically from parents as well as from the environment yields rank-abundance curves that fit a log-normal distribution (27), which also does not match our results. Rather, the fit determined on the basis of a power law or modified power-bend curve provided a closer match of the data. Almost all gut samples had a long tail of rare species with many OTUs and few read counts (Fig. 4B to E), which has previously been reported for marine microbial communities (28) and is characteristic of bilaterian gut communities as well. Such long tails of rare species are inconsistent with the predictions of neutral theory.

### Influence of data processing on the conclusions drawn.

Several approaches were taken to assess the influence of sequencing error on our conclusions. Chimera filtering was not applied to the data initially due to the difficulty of identifying chimeras involving pairs of previously unstudied 16S rRNA gene sequences. To test for possible confounding by chimeras, we applied chimera filtering using ChimeraSlayer and USEARCH to our gut microbiome samples (29, 30). ChimeraSlayer found 0.16% potential chimeras and USEARCH found 3.4% potential chimeras, suggesting that chimera formation did not strongly influence our conclusions.

To test for possible effects of sequencing error, we denoised our data set using the filtering program DADA2 (31). DADA2 assumes that all sequence variants represented by single reads are products of error and removes them and clusters sequences into biological sequence variants based on the estimated probability of a read being a sequencing error. DADA2 yielded 84,259 sequence variants compared to the 521,510 OTUs yielded by the processing strategy described above. The shapes of species rank abundance curves were altered by DADA2 filtering such that the Poisson lognormal model was now favored over the power law models (Fig. S6A); however, data both before and after filtering did not match predictions of the neutral model. The species-area relationship was still observed after DADA2 filtering (Fig. S6B). Thus, our main conclusions were robust with respect to the data filtering strategy chosen.

10.1128/mBio.00319-18.6 FIG S6  (A) Fit of models to rank-abundance curves after denoising of sequence reads with DADA2. The abundances of DADA2 sequence variants from samples were assessed for their fits to community models (truncated to observations totaling >1 to account for DADA2’s removal of singletons) (columns) using Akaike information content (AIC) and the AIC averaged within each species (rows). Within each row, the best-fitting models (i.e., lowest AIC) appear red, with the color code showing the difference for each model from the minimum AIC. White coloring indicates a model convergence failure. (B to E) Empirical rank-abundance curves and comparisons to model best fits are shown for single samples from human (B), right whale (C), tiger shark (D), and cricket (E). (B) Comparison of animal weight to the number of DADA2 filtered sequence variants. Points indicate the average animal weights and the expected number of DADA2 filtered sequence variants after rarefaction to 500 reads for each species. Points are colored by host phylogenetic class as described for Fig. 2. The line indicates a linear regression of the log of the number of sequence variants on log host weight, and shading indicates the 95% confidence interval for the regression. Download FIG S6, PDF file, 1 MB. Copyright © 2018 Sherrill-Mix et al. 2018 Sherrill-Mix et al. This content is distributed under the terms of the Creative Commons Attribution 4.0 International license.

## DISCUSSION

Here we sought to test the hypothesis that the bilaterian gut microbiota is assembled by selective forces, resulting in communities diverging from the predictions of neutral colonization. For this, we assembled a broad sample set, ranging from small insects to baleen whales. Although myriad factors affect microbial community structure in each organism, our survey establishes several generalizations. The structure of gut microbial communities commonly partitioned with host taxonomy, indicating that gut microbiome structure can be preserved over long evolutionary time scales. Species-area analysis showed an increase in species richness with gut size, supporting the ideas that bacterial populations in larger guts are less likely to become extinct due to chance fluctuations and that larger guts may have more types of environmental niches. Consistent with these findings, predictions from quantitative models based on ecological neutral colonization did not match most samples, supporting an alternative model in which selection drives gut microbial community assembly. We also report numerous detections of highly divergent bacterial taxa, which can help target future studies to characterize global bacterial diversity.

Our finding of a positive association between bacterial species richness and host size suggests that mechanisms driving classic species-area relationships in island biogeography (1–3, 32) are also at play in gut microbial communities. Previous efforts to detect species-area relationships in the gut microbiota were mixed (4, 5)—the authors of the study that did not report a significant slope used data processed in multiple different ways from previous studies and themselves pointed out that this could have affected the outcome (4). For insects, estimates of microbial community size per individual can range from 10^9^ bacterial cells to undetectable numbers (33); thus, fluctuations leading to extinction may be possible for the sparser communities. For larger organisms such as vertebrates, numerous studies have emphasized the heterogeneous anatomy of the gut at many spatial scales such that greater heterogeneity in microbial niches would be expected (9). Consistent with this, we found that the proportion of anaerobes was significantly higher in guts of larger animals. Previous data indicated that the lumen of the gut of mice is relatively anaerobic, whereas gut tissue is more oxygenated, and that this results in enrichment of numbers of anaerobic bacteria in the lumen and of aerobic bacteria near the gut wall (34). For bilaterians of smaller sizes, oxygen may permeate more readily throughout the gut, resulting in predominance of aerobes and reduced overall bacterial species richness. For larger bilaterians, the combination of aerobic and anaerobic environments may result in formation of bacterial communities that are richer in species.

Our values for the slope of the species-area relationship correspond to slopes that are shallower than those typical of classical studies of island populations, which typically range from 0.12 to 0.35 (32). This shallower slope may indicate that animal guts are not as insular as islands with more-frequent (potentially low-abundance) immigrations from the environment/conspecifics or with a slower accumulation of habitat heterogeneity in large guts than on large islands. A potential technical issue which might have contributed to our findings is that smaller animals were often studied as dissected guts, potentially resulting in representation of a greater number of different niches than were present in fecal samples from larger animals.

Several aspects of our data argue against neutral (nonselective) processes in colonization of the bilaterian gut. The profiles of species-abundance curves display long tails (Fig. 4)—representative of the presence of many species at low abundance—which are not predicted by neutral theory. For marine organisms, abundant seawater lineages were scarce in gut samples, highlighting likely selection by the gut environment (see Table S2 in the supplemental material). For certain insects, some of the gut microbiotas have been reported to resemble local environmental taxa and to differ among individuals of the same species (33), suggesting possible neutral colonization from the environment. However, for the great majority of insects studied here, gut community rank-abundance curves did not show the profile expected for neutral colonization. For mammals, attempts to introduce new bacterial strains into the gut generally work poorly in a colonized host (35-38), consistent with gut bacterial communities adapting to their environments and so resisting invasion by new strains.

Numerous further theories have attempted to explain the shapes of species-abundance curves (39). Unfortunately, while interesting, these models rarely make unique predictions and so do not specify unique assembly mechanisms that can be applied here. Several lines of evidence have suggested that communities more open to immigration have a higher proportion of rare species (39), which provides a candidate mechanism contributing to the long tails typically observed for gut microbial species-abundance curves.

Despite the geographic dispersal of bilaterian individuals, we found that members of the same species usually resembled each other with respect to the characteristics under consideration more than they resembled members of other species. This supports the inference that dispersal of microbes between individuals is usually not limiting. Several mechanisms are known to assist transfer of microbiota between generations within a species. Vertical inheritance is well known for *Wolbachia* in invertebrates (40). Some insect mothers smear fecal material on newly deposited eggs (40). Cohabitation is associated with sharing bacterial lineages among social insects (33), laboratory mice (11), and humans and their dogs (41). Transmission via breastfeeding has been proposed to mediate human mother-to-infant transmission (42). Thus, the recurrent patterns of microbial community membership within host species suggest that dispersal of microbes is usually not a barrier to gut community construction.

This study had several limitations. The use of 16S rRNA gene tags provides a tractable window on community structure for a sample set that includes tissue specimens, but additional studies using shotgun metagenomic sequencing could yield richer (though quite complex) data. Although a large number of specimens and species were studied, our sampling scheme was opportunistic and the coverage of *Bilateria* uneven. Going forward, it would be valuable to target specific animal groups with focused questions, for example, to investigate the influences of diet and lifestyle (laboratory housed, domestic, or wild) and associations with humans.

In summary, these data suggest that neutral assembly models cannot explain the structure of the bilaterian gut microbiome and that niche selection is a likely driver. Larger animals harbor richer bacterial communities, potentially a consequence of the presence of more-diverse niches in gut, including an increase in anaerobic habitat, and greater resistance to stochastic extinction due to larger sizes of microbial populations. This work provides baseline data for understanding the structure and dynamics of the global bilaterian microbiome and provides a point of departure for additional studies. Going forward, the data and analytic methods presented here may be useful in assessing colonization mechanisms in human disease states (43, 44) and in evaluating the invasion of human-associated bacteria into global ecosystems.

## MATERIALS AND METHODS

### Sources of samples.

Sources of samples are listed in Table S1 in the supplemental material, and additional sample information is available in the supplemental material.

### DNA preparation.

DNA from all samples was isolated in a sterile class II laminar flow hood. Due to differences in starting material, DNA was purified using three methods. First, DNA was isolated from fecal samples using a Mo Bio PowerSoil HTP 96 DNA isolation kit (Mo Bio Laboratories, Inc., Carlsbad, CA). Sample inputs weighed between 0.0085 g (gerbil pellet) and 1.6408 g (gut contents of the large intestine of a sandbar shark). Samples were incubated for 10 min at 70°C and homogenized for 20 min with a Qiagen TissueLyser II instrument, and DNA was purified per the manufacturer’s protocol. Second, to reduce background, DNA from low-biomass samples (primarily the dissected gut tissue from smaller organisms) was purified using a Qiagen DNeasy UltraClean microbial kit (Qiagen, Hilden, Germany), in single tubes, per the protocol of the manufacturer, including the recommended bead-beating step. Finally, DNA from whale feces was isolated with a PSP Stool DNA Plus kit (Stratec Biomedical, Berlin-Buch, Germany). Samples were homogenized in PSP stool DNA stabilization buffer using the TissueLyser II instrument and incubated at 95°C, and DNA was purified per the manufacturer’s protocol. All DNA was stored at −20°C.

### Quantification of 16S rRNA gene copies using qPCR.

16S rRNA gene copies were quantified by the amplification of the V1V2 region of the 16S rRNA gene by quantitative PCR (qPCR). Dilutions (1:4) of each DNA sample were used in reaction mixtures (total volume, 25 µl) and measured in triplicate. Primer and probe sequences and amplification conditions are described in Table S3 and reference 45.

10.1128/mBio.00319-18.9 Table S3  Sequences of DNA oligonucleotides used in this study. Download Table S3, XLSX file, 0.01 MB. Copyright © 2018 Sherrill-Mix et al. 2018 Sherrill-Mix et al. This content is distributed under the terms of the Creative Commons Attribution 4.0 International license.

### PCR amplification of the V1V2 region of the bacterial 16S rRNA gene for Illumina sequence analysis.

For each sample, the V1V2 region of the bacterial 16S rRNA gene was amplified with Golay bar-coded universal primers 27F and 338R (Table S3) (41, 46, 47). PCRs were performed in triplicate with 5 µl DNA, 7.21 µl PCR-grade water, 2.5 µl 10× buffer II, 0.19 µl *Taq*, 5 µl of the forward primer (2 µM), and 5 µl of the reverse primer (2 µM). PCRs were prepared in a PCR clean room using an EpMotion 5075 liquid handling workstation (Eppendorf, Hamburg, Germany) and run in quadruplicate on an Applied Biosystems GeneAmp PCR 9700 system (Thermo Fisher Scientific Inc., Waltham, MA) under the following cycling conditions: initial denaturation at 95°C for 5 min; 30 cycles of denaturation at 95°C for 30 s, annealing at 56°C for 30 s, and extension at 72°C for 90 s; and a final extension at 72°C for 8 min. Reaction replicates were pooled into libraries and purified using Agencourt AMPure XP beads (Beckman Coulter, Inc., Indianapolis, IN) per the manufacturer’s protocol. The purified PCR products were then pooled and sequenced using an Illumina MiSeq platform. qPCR quantification of 16S rRNA gene copies was used to ensure that a minimum of 1,000 16S rRNA gene copies were input into each reaction. To pass our quality filter, samples were also required to contain at least 1,000 Illumina sequence reads.

### Analytical methods.

Sequence data was processed using QIIME 1.9.1 with default parameters (48). OTUs were selected by clustering reads at 97% sequence similarity. Taxonomic assignments were generated by comparison to the Greengenes reference database (49). The consensus taxonomy assignment implemented used the most detailed lineage description shared by two of the top three best-matching reference sequences in the Greengenes database. We mostly used data without rarefaction in order to maximize the amount of sequence information available. In cases where the amount of sequence analyzed could have affected the outcome, as in the species-area analysis, we did use rarefied data (for each sample, we calculated the number of OTUs expected to be observed for 100 reads). Additional analytical methods can be found in the Appendices.

### Data availability.

Sequences from this study are available at the NCBI SRA (https://www.ncbi.nlm.nih.gov/Traces/study/?acc=SRP115877).

## APPENDIX

### Additional sample information.

Most of the information on samples studied is provided in Table S1. Some additional data is as follows.

Feces samples from healthy human individuals were obtained from a previous study approved by the Perelman School of Medicine at the University of Pennsylvania Institutional Review Board (50). Six rhesus macaques (*Macaca mulatta*) with no history of gastrointestinal disease were sampled at the Tulane National Primate Research Center. Fecal samples were collected from stainless steel collection pans below the animals’ cages. Fecal samples (*n* = 111) from western (*P. t. verus*, *n* = 1), Nigeria-Cameroonian (*P. t. ellioti*, *n* = 1), central (*P. t. troglodytes*, *n* = 5) and eastern (*P. t. schweinfurthii*, *n* = 33) chimpanzees and from western (*G. g. gorilla*, *n* = 3) and eastern (*G. b. graueri*, *n* = 2) lowland gorillas, as well as from bonobos (*Pan paniscus*, *n* = 66), were obtained from existing specimen banks (51–56). Except for four specimens (CCptv01, SYptt43, TCptt98, and CFpts01) that were obtained from sanctuary chimpanzees, all other fecal samples were collected from wild-living ape populations at remote forest sites (Table S1) (Fig. S1D). All samples were obtained noninvasively, preserved (1:1 [vol/vol]) in RNAlater, transported at ambient temperatures, and stored at −80°C. Fecal DNA was extracted using a QIAamp Stool DNA minikit (Qiagen). All specimens were subjected to host mitochondrial DNA analysis to confirm their species and subspecies origin (51–58). In the analysis, each of the four chimpanzee subspecies was treated as a separate species. Samples were also obtained from two mandrills from the Tchimpounga sanctuary and two habituated sooty mangabeys from the Tai Forest. Sampling sites are shown in Fig. S1D. All samples were obtained with approval from the respective governments as previously described (51–56) and were shipped in compliance with regulations from the Convention on International Trade in Endangered Species of Wild Fauna and Flora and country-specific import and export permits. Fecal pellets from healthy laboratory mice were collected at the Perelman School of Medicine at the University of Pennsylvania (protocol no. 803408). Right whale samples were collected under Fisheries and Oceans Canada license no. 325842 (59), the fin whale sample was collected under NOAA permit no. 16325, and the humpback samples were provided by Jan Straley, collected under NOAA permits 474-1700-02 and 14122. Shark specimens were collected as part of commercial fishing activities regulated by the National Marine Fisheries Service Federal Management Plan for Atlantic Tunas, Swordfish, and Sharks.

In instances where there was no available expelled fecal material, such as for some insects, samples were obtained by gut dissection of the frozen individuals. Each individual was weighed, washed with 70% ethanol, and rinsed with phosphate-buffered saline (PBS). Individuals were then placed on a sterile petri dish, and the gut was removed with sterile needle nose tweezers.

### Additional analytic methods.

The aerobic or anaerobic status of bacterial taxa was copied by hand from reports in Bergey’s Manual of Systematics of Archaea and Bacteria (60). A detailed description of all annotations used is provided in Table S4. The anaerobic or aerobic status was scored for each bacterial lineage, starting with the lowest-ranking taxon and moving on to higher-ranking taxa. Aerobic or anaerobic status was assigned to taxa above the rank of genus if listed in the description of the taxon or if >99.9% of reads assigned to lower-ranking taxa observed in this study had a consistent status (further details are provided in Table S4).

10.1128/mBio.00319-18.10 Table S4 Bacterial annotations used to score microbial OTUs as anaerobic, facultatively anaerobic, and aerobic. Download Table S4, XLSX file, 0.05 MB. Copyright © 2018 Sherrill-Mix et al.2018Sherrill-Mix et al.This content is distributed under the terms of the Creative Commons Attribution 4.0 International license.

Phylogenetic trees were inferred from the OTU data using FastTree (61). Similarity between samples was assessed by weighted and unweighted UniFrac distance (62, 63). Data files from QIIME were analyzed in the R environment for statistical computing. Phylogenetic distances were retrieved from TimeTree (24), using a closely related proxy species if necessary. We used the Adonis function from the vegan package to perform PERMANOVA.

Maximum likelihood estimates for various species abundance distributions were fitted using the sads R package (64) with functions modified slightly for additional robustness (e.g., multiple starting values for optimization and calculations on log values to avoid numeric overflow). Fitted models included the broken stick model (65); log series (66, 67); neutral metacommunity zero-sum multinomial (6, 66, 68); zeta, power, and power bend (69); Poisson lognormal (70); lognormal (71); Weibull; and negative binomial, gamma, and geometric distributions. Models were compared using the Akaike information criteria to control for overfitting in models with additional parameters.

We obtained additional data from previously published studies on the microbiome of insects (12), birds (13), primates (14), myrmecophagous mammals (15), fish (16), diverse mammals sequenced using the 454 method (17), whales and other mammals (18), and diverse mammals sequenced using the Illumina method (19). The whale data included both 454 sequencing and Illumina sequencing data, which were treated separately. The insect data contained unpaired reads from the 5′ and 3′ ends of the amplicon, which were treated separately. Thus, we had data from 8 additional studies broken into 10 data sets.

To estimate the species-area relationship in animal guts, we fit a Bayesian regression model of the rarefied OTU counts observed in each animal gut in relation to the mean weight of the hosts using Stan (72). This model included samples from our own data set and the 10 additional data sets. As extraction and amplification methods, amplicon targets, and sequencing technologies differed between data sets, we allowed each data set its own offset. To compare the data from the studies whose results are shown in Fig. 3, we subtracted the mean of the estimated posterior distribution for these offsets. The model assumed that the log rarefied OTU count, count_*i*_, in sample *i* were normally distributed as follows:

$${\text{count}}_{i}=N(\mu_{i},\sigma_{\text{dataset}}{}_{{}_{i}})$$

where dataset_*i*_ is the data set that sample *i* came from, σ_*x*_ is the estimated variability in data set *x*, and μ_*i*_ is given by following equation:

$$\mu_{i}={\text{offset}}_{{\text{dataset}}_{i}}+\beta {\text{weight}}_{{\text{species}}_{i}}+\theta_{{\text{species}}_{i}}$$

Where species_*i*_ is the host species for sample *i*, offset_*x*_ is the offset for data set *x*, weight_*x*_ is the log weight for species *x*, β measures the relationship between weight and OTU count, and θ_*x*_ is the mean offset for species *x* from a linear weight-count relationship. Species θ values were given a normal prior with mean equal to 0 and a separate variance value for each phylogenetic class. Variance parameters were given a gamma(1,1/10) prior.

Reads that were annotated by Greengenes as coming from chloroplast genomes were removed. Samples that contained fewer than 1,000 sequence reads were removed. We investigated potential chimeric reads generated by amplification and sequencing using ChimeraSlayer and USEARCH (29, 30). ChimeraSlayer uses BLAST alignment against representative aligned sequences to identify potential chimera parents. USEARCH performs *de novo* chimera detection based on abundances as well as reference-based chimera detection for raw sequence reads. ChimeraSlayer found 1,265 potential chimeras of 779,732 representative OTU sequences. There was little association between these chimeras and singleton OTUs (95% confidence interval of odds ratio [CI OR], 0.84 to 1.05; Fisher’s exact *P* = 0.28) or reads unassigned to the Greengenes database (95% CI OR, 0.85 to 1.13; Fisher’s exact *P* = 0.8). USEARCH found 776,531 potential chimeras among 22,538,324 total sequence reads. Among USEARCH chimeras, there was a slight depletion of singleton reads (95% CI, 0.69 to 0.70; Fisher’s exact *P* < 10^−6^) and of reads unassigned to Greengenes (95% CI, 0.59 to 0.60; Fisher’s exact *P* < 10^−6^) but the association was modest.

Code used in this analysis is archived on Zenodo at https://doi.org/10.5281/zenodo.1194331.
