# Supplementary figures and images for: Allometry and Ecology of the Bilaterian Gut Microbiome
Source: mBio. 2018 Mar 27;9(2):e00319-18. doi: 10.1128/mBio.00319-18 (PMC5874926; doi:10.1128/mBio.00319-18)

Figure S1  
A

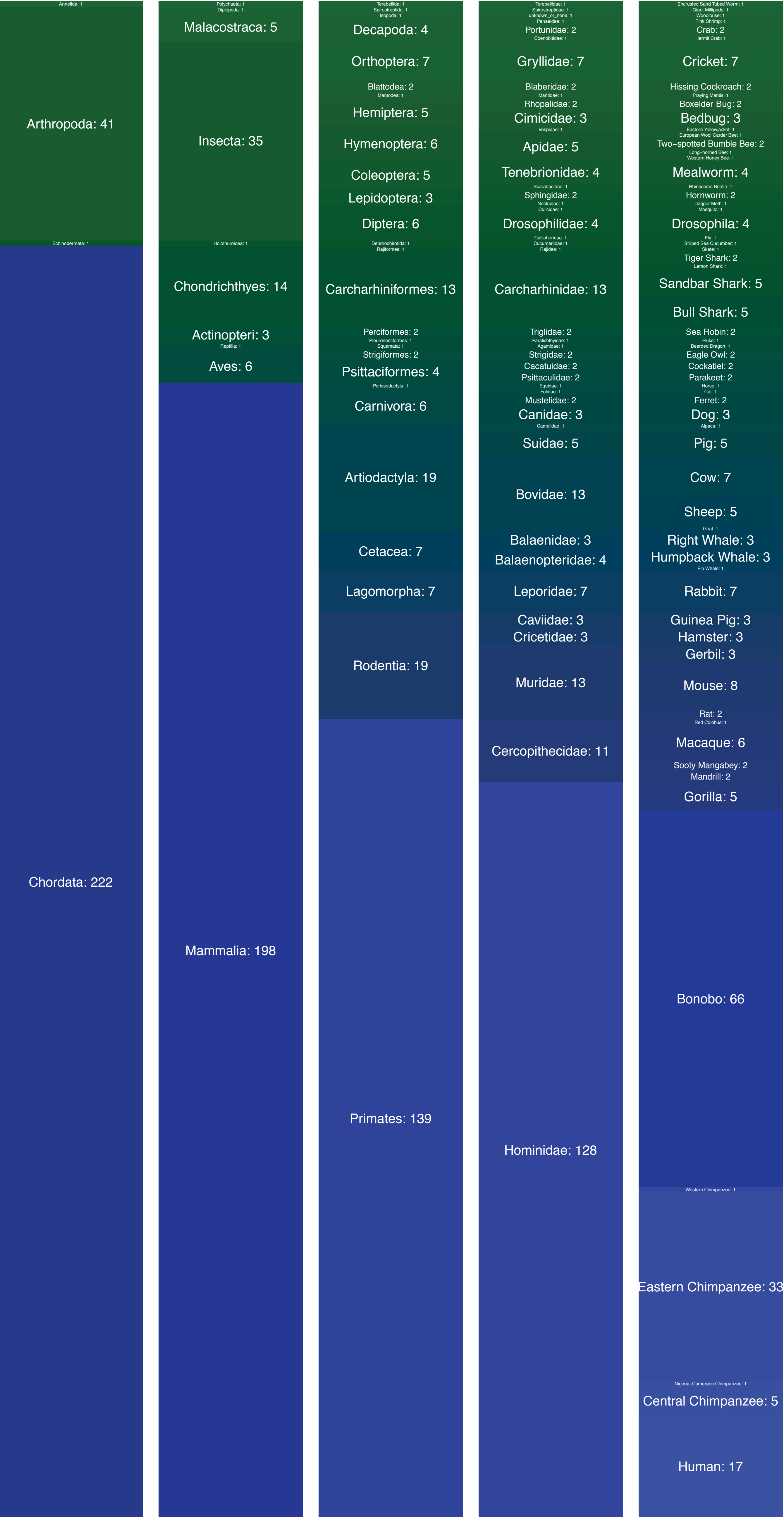

B

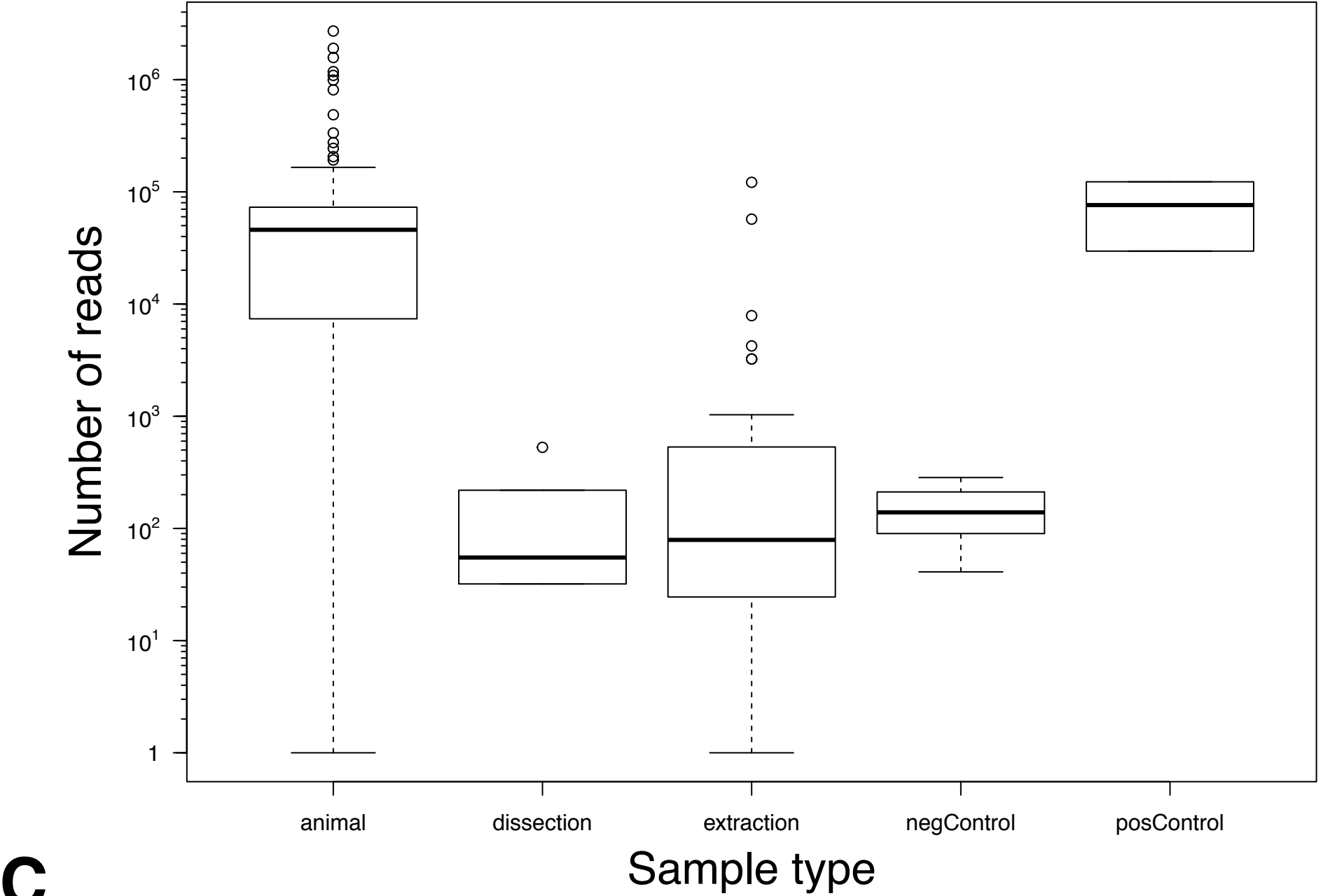

C

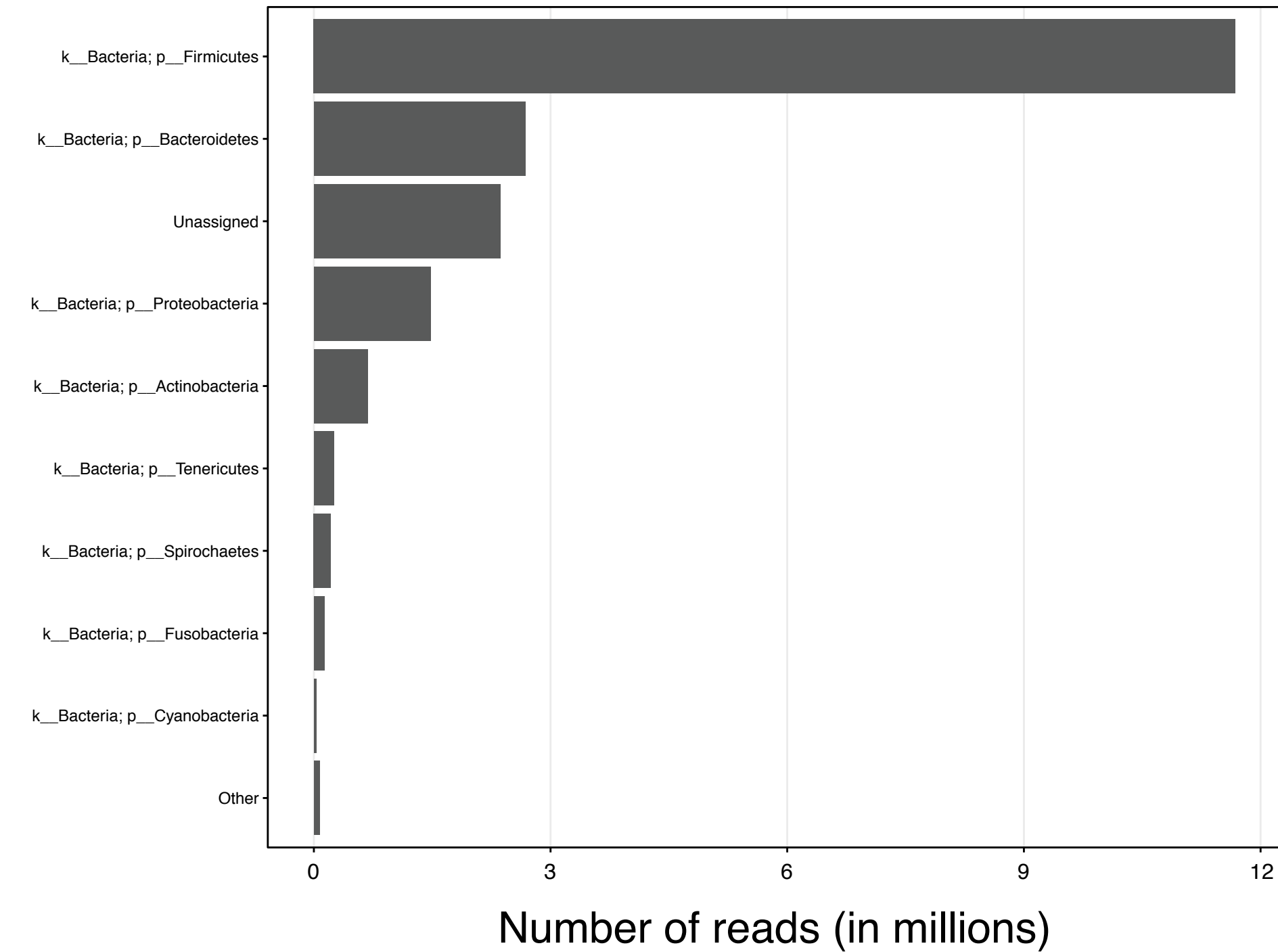

D

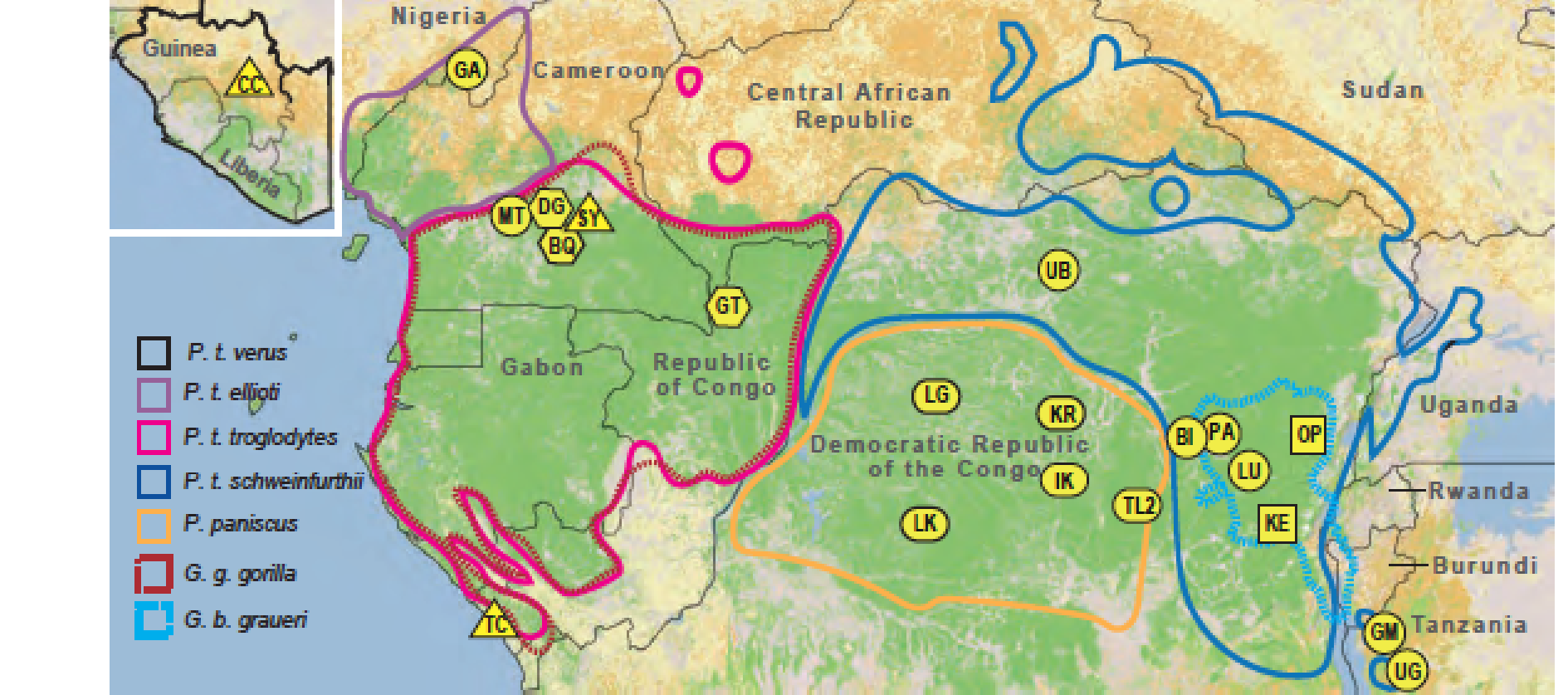

Supplement: FIG S1 [file mbo002183790sf1.pdf]

Figure S3  
A

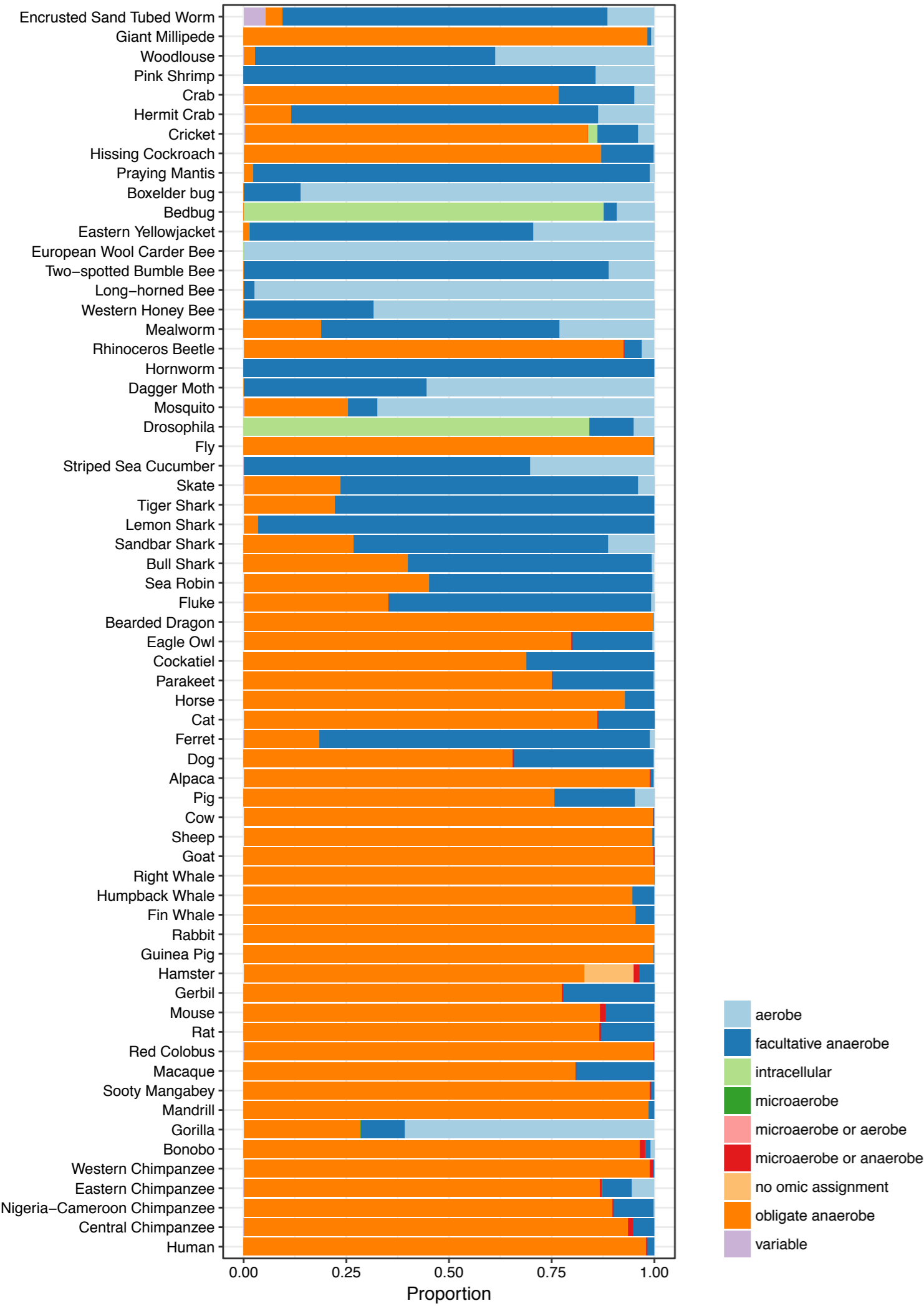

B

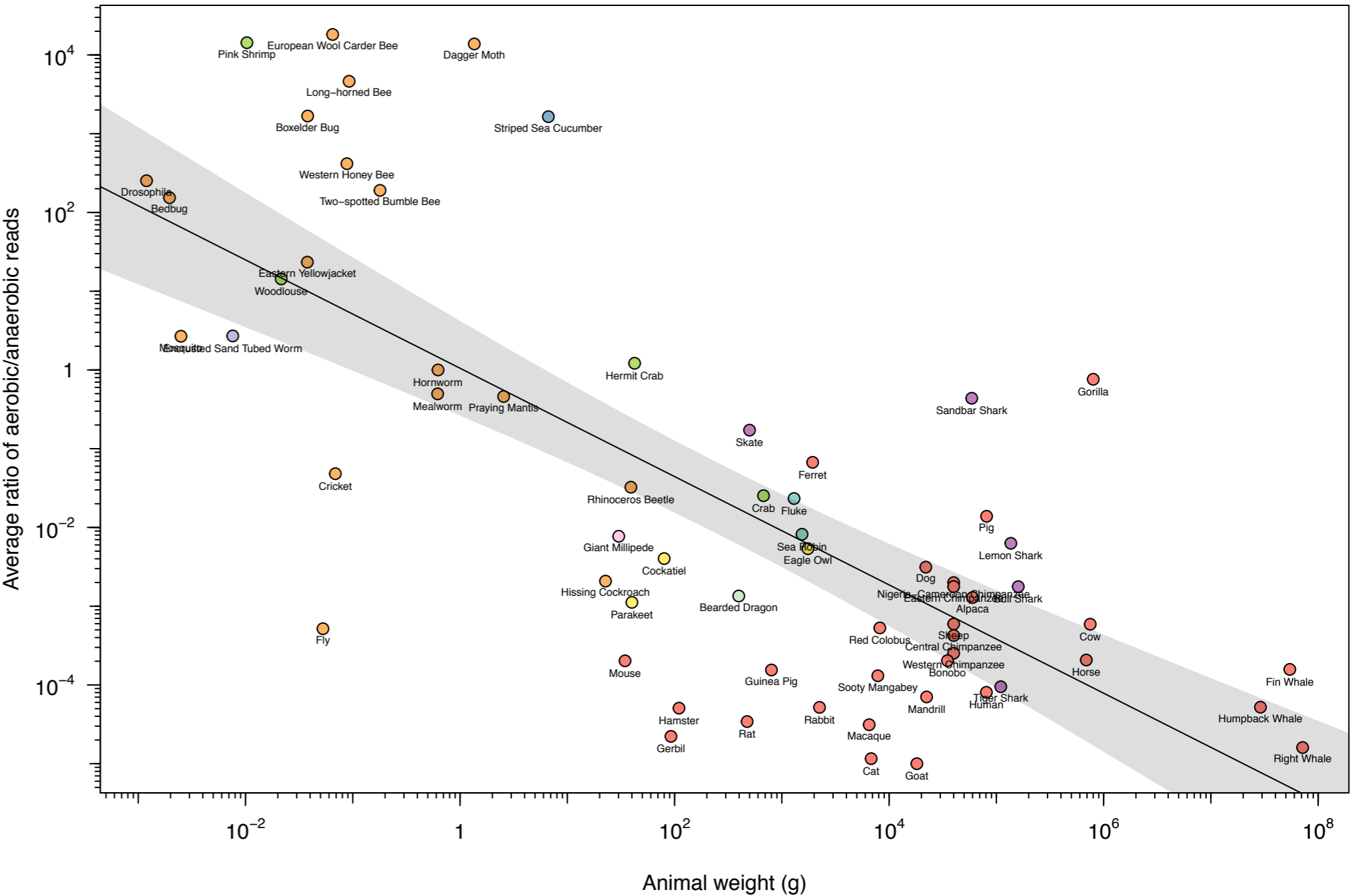

Supplement: FIG S3 [file mbo002183790sf3.pdf]

Figure S4

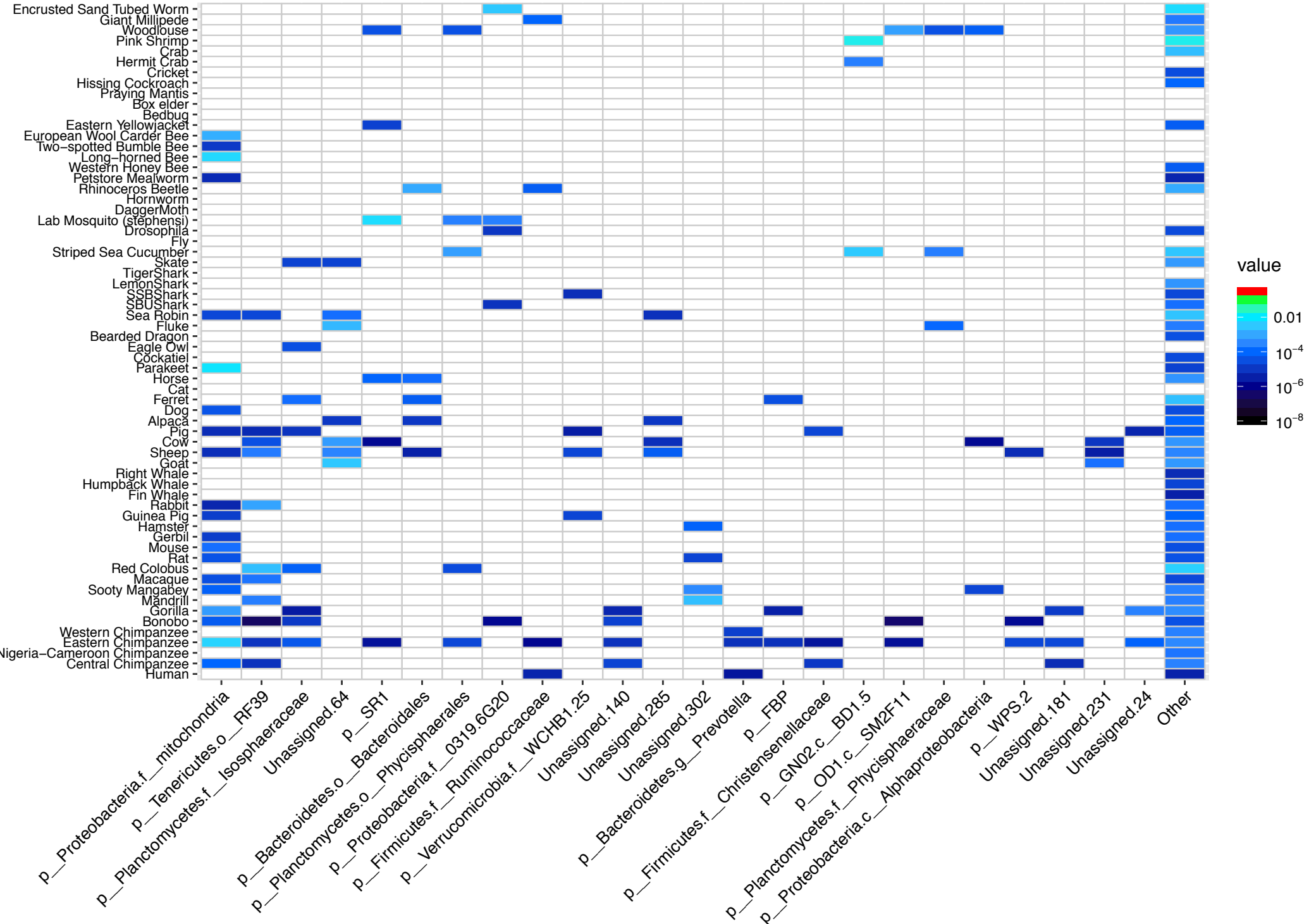

Supplement: FIG S4 [file mbo002183790sf4.pdf]

Figure S5

A

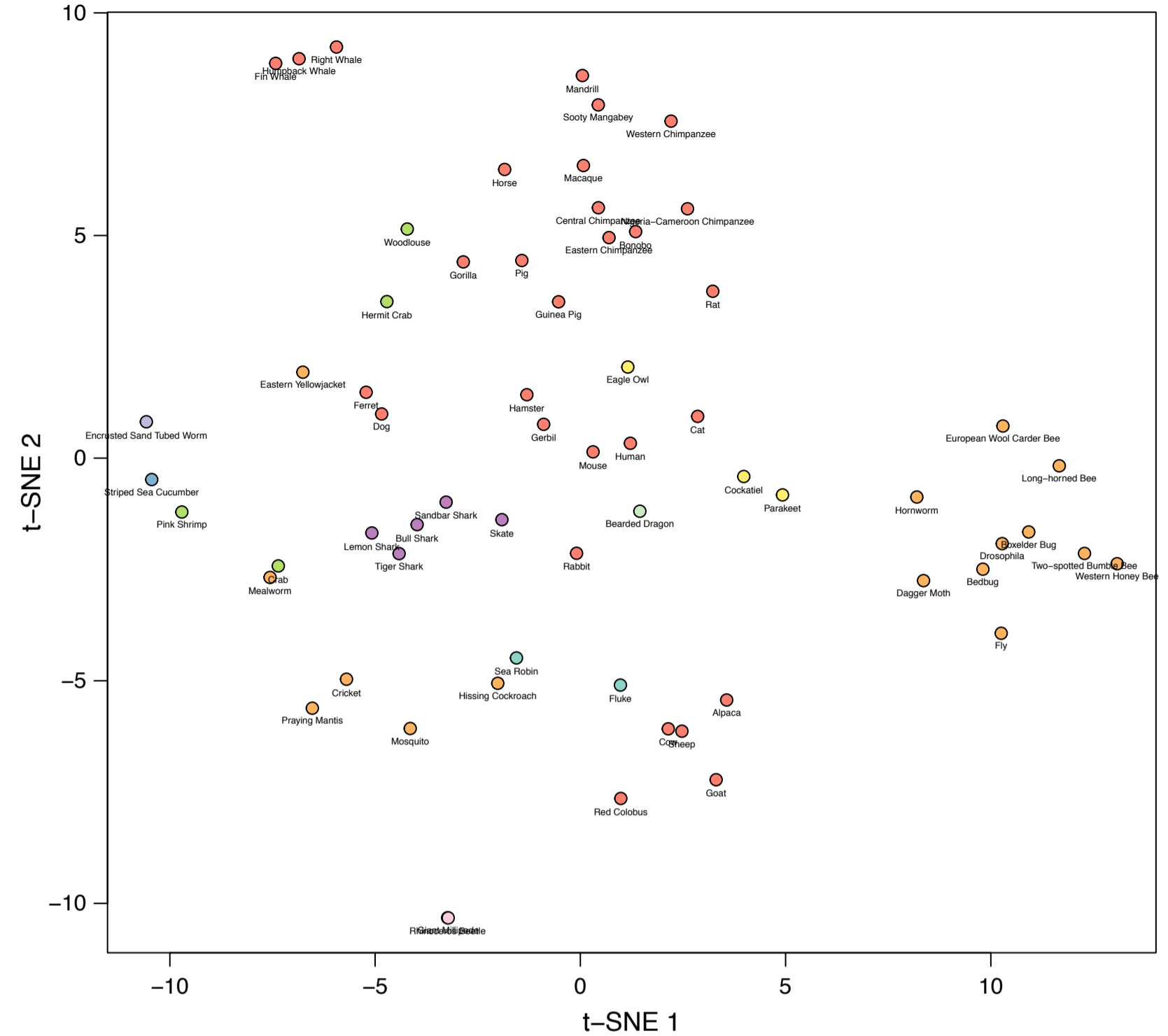

B

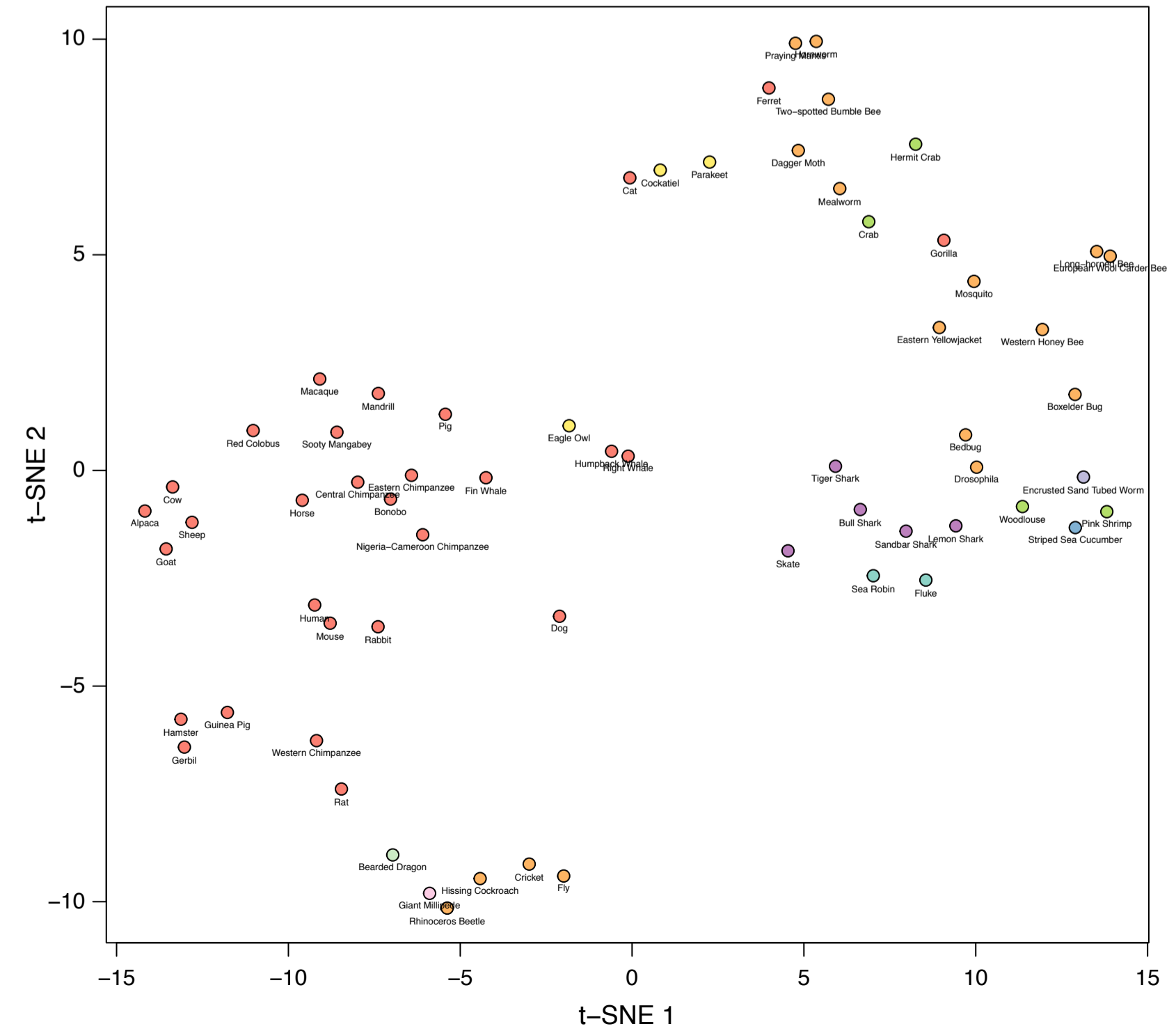

Supplement: FIG S5 [file mbo002183790sf5.pdf]

**A**

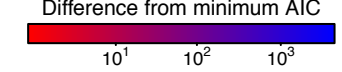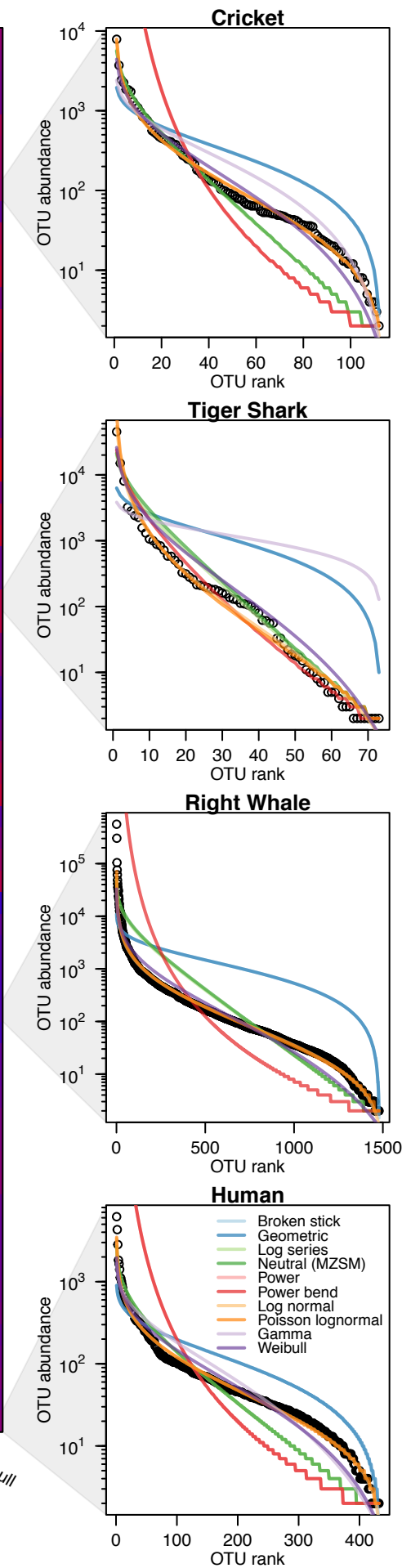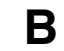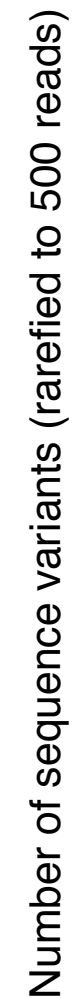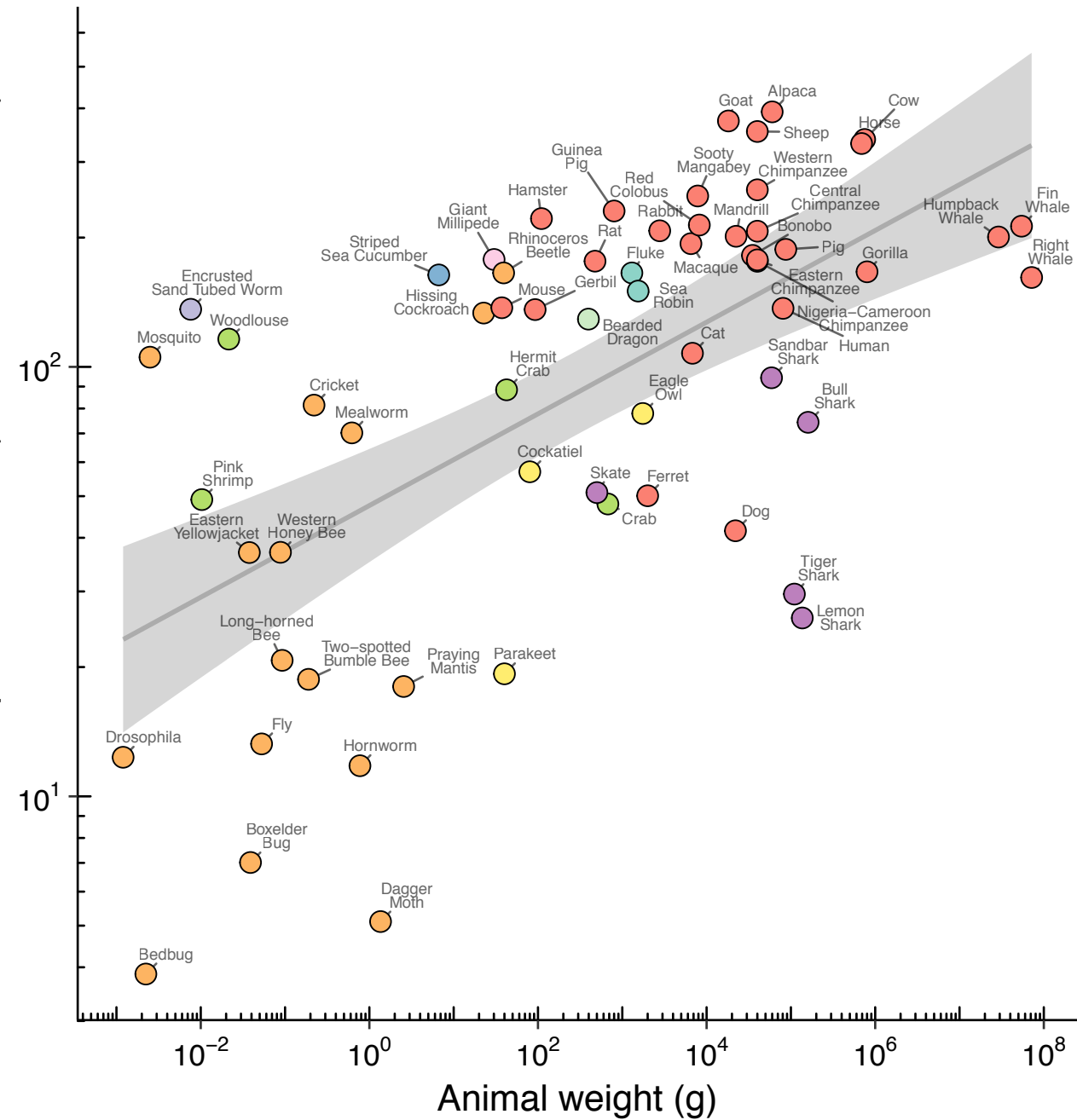

Supplement: FIG S6 [file mbo002183790sf6.pdf]
