# Supplementary material for: Allometry and Ecology of the Bilaterian Gut Microbiome
Source: mBio. 2018 Mar 27;9(2):e00319-18. doi: 10.1128/mBio.00319-18 (PMC5874926; doi:10.1128/mBio.00319-18)
Supplement: FIG S2 [file mbo002183790sf2.pdf]

## Figure S2

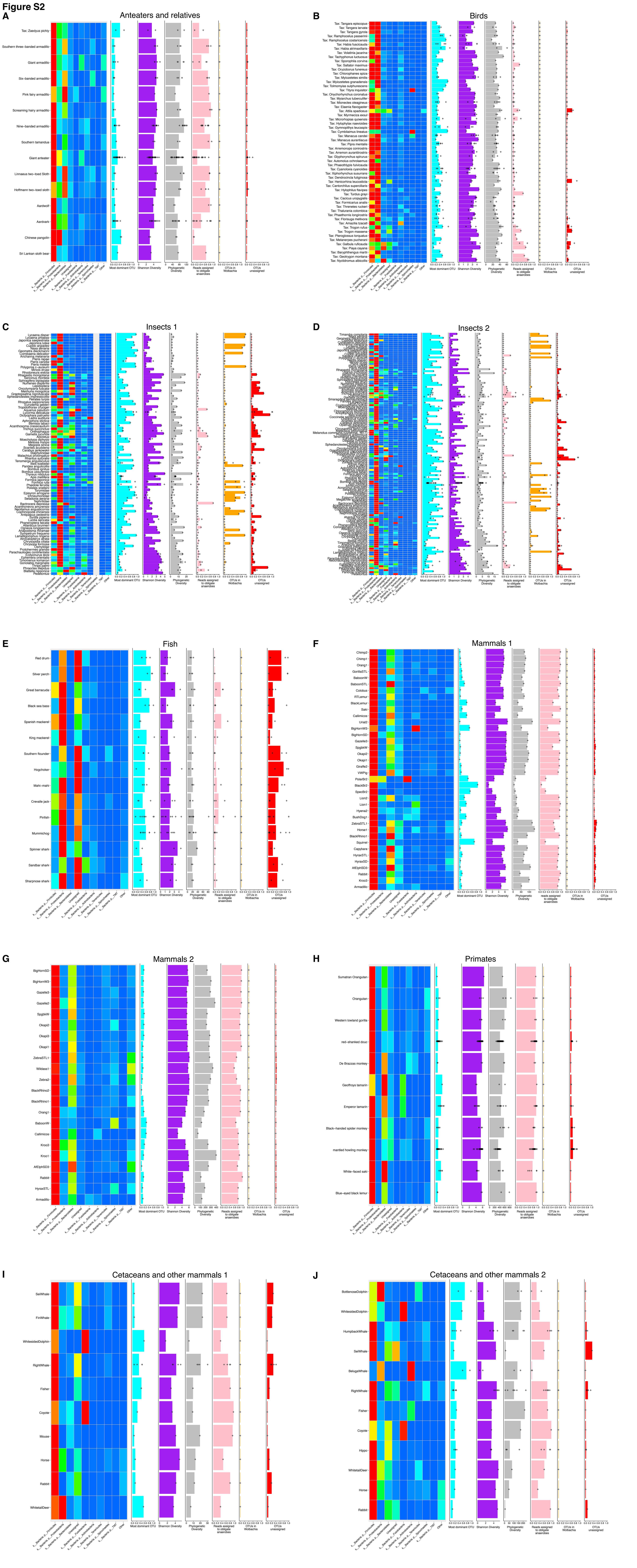
A
Anteaters and relatives
B
Birds
C
Insects 1
D
Insects 2
E
Fish
F
Mammals 1
G
Mammals 2
H
Primates
I
Cetaceans and other mammals 1
J
Cetaceans and other mammals 2
Most dominant OTU
Shannon Diversity
Phylogenetic Diversity
Reads assigned to obligate anaerobes
OTUs in Wolbachia
OTUs unassigned
